# Ag_12_[Ge_9_(Hyp)_2_]_6_ An Intermetalloid Cluster with Bis‐Silylated Ge_9_ Units

**DOI:** 10.1002/anie.202521991

**Published:** 2025-12-04

**Authors:** Kevin Woern, Claudio Schrenk, Eric Juratti, Andreas Schnepf

**Affiliations:** ^1^ Institute of Inorganic Chemistry University of Tuebingen Auf der Morgenstelle 18 72076 Tübingen Germany; ^2^ Institute of Physical and Theoretical Chemistry University of Tuebingen Auf der Morgenstelle 18 72076 Tübingen Germany

**Keywords:** Germanium, Intermetalloid Cluster, Silver

## Abstract

We present the synthesis and characterization of the novel intermetalloid cluster compound Ag_12_[Ge_9_(Hyp)_2_]_6_, exhibiting a silver core surrounded by bis‐silylated Ge_9_‐clusters. The new intermetalloid cluster has an Ag_12_ metal cluster core with silver atoms in different oxidation states. To the silver core six [Ge_9_(Hyp)_2_] units are coordinated, acting as ligands. The [Ge_9_(Hyp)_2_] units are arranged octahedrally around the Ag_12_ core, leading to a variety of Ag‐Ge bonds and contacts. The cluster is a first example of an intermetalloid cluster, exhibiting a silver core, shielded by [Ge_9_(Hyp)_2_] units.

## Graphical abstract

The reaction of K_2_Ge_9_(Hyp)_2_ (Hyp = Si(SiMe_3_)_3_) with phosphine stabilized silver halides gives the new intermetalloid cluster Ag_12_[Ge_9_(Hyp)_2_]_6_ exhibiting a novel Ag_12_ core surrounded by six [Ge_9_(Hyp)_2_] units acting as ligands. Within the Ag_12_ core two different kinds of silver atoms are present underlining the intermetalloid character of the cluster.

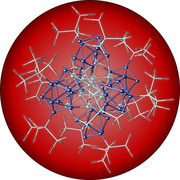

Nine atomic germanium clusters are known for a long time now.^[^ ^1^ ^]^ The most prominent example is the polyanion Ge_9_^4−^, which can be found, e.g., in the Zintl phase K_4_Ge_9_.^[^ ^2^ ^]^ Beside the chemistry of these intermetallic phases our working group developed the synthesis of the metalloid cluster Ge_9_(Hyp)_3_^−^ (Hyp = Si(SiMe_3_)_3_) starting from a Ge(I)Br solution about 20 years ago.^[^ ^3^ ^]^ In both cases the cluster core can either be described as a tricapped trigonal prism or as a monocapped square antiprism.^[^ ^4^ ^]^ Beside these structural similarities, it took until 2012, since Sevov and coworkers were able to synthesize Ge_9_(Hyp)_3_^−^ directly from the Zintl phase K_4_Ge_9_ by reaction with Hyp‐Cl.^[^ ^5^ ^]^ The huge chemical potential of those Ge_9_ cages was already shown by the formation of dimers,^[^ ^6^ ^]^ trimers,^[^ ^7^ ^]^ tetramers,^[^ ^8^ ^]^ and even infinite chains^[^ ^9^ ^]^ through the oxidative coupling of Ge_9_^4−^ units. It is even possible to use the dimers and trimers for follow up reactions, as shown by Sun et al. who were able to insert a NbCp_2_ fragment into them.^[^ ^10^ ^]^ As a highlight, it was shown that a new modification of the element germanium (Ge‐cF136) can be obtained through oxidation of Ge_9_^4−^ within an ionic liquid.^[^ ^11^ ^]^ When oxidized with a milder oxidation reagent namely Co(dppe)Cl_2_ it is possible to get the cluster Ge_24_^4−^ which consists of two Ge_9_ units connected by a central Ge_6_ compound.^[^ ^12^ ^]^ In addition, a variety of coupling reactions involving transition metals are possible. For example, the reaction of Ge_9_^4−^ with elemental mercury leads to a mercury‐coupled polymer.^[^ ^13^ ^]^ The reaction with Ni[COD]_2_ (COD = cyclooctadiene) results in a compound containing a linear nickel trimer enclosed within two Ge_9_ cages.^[^ ^14^ ^]^ The reaction of Ge_9_^4−^ with the phosphine stabilised gold halide (Ph_3_P)AuCl leads to a compound containing two Ge_9_ units, both of which are bonded to a central triangular Au_3_ unit.^[^ ^15^ ^]^ The same reaction, but with different conditions, gives a larger compound consisting of a unique Ge_45_ unit and, again, a central triangular Au_3_ unit.^[^ ^16^ ^]^

Also the metalloid cluster Ge_9_(Hyp)_3_^−^ can be used in subsequent reactions, as shown at first by our group by synthesizing [Ge_9_Hyp_3_‐TM‐Ge_9_(Hyp)_3_]^x^ (TM:, e.g., Cu, Ag, Au with *x* = 1‐ or Zn, Cd, Hg with *x* = 0).^[^ ^17^, ^18^, ^19^ ^]^ After Sevov's approach and the fact that now one is no longer depending on the unique chemical Ge(I)Br, plenty of reactions were done with this metalloid cluster Ge_9_(Hyp)_3_^−^ using it as a building block in coordination chemistry. Here compounds of early^[^ ^20^ ^]^ and mid transition metals,^[^ ^21^ ^]^ as well as double‐ or triple‐coordinated transition metals to one or two Ge_9_(Hyp)_3_ units could be found.^[^ ^22^, ^23^ ^]^ Even an oxidation similar to the formation of Ge‐cF136 is possible, ending up at isomers of the, to date largest metalloid germanium cluster Ge_18_(Hyp)_6_.^[^ ^24^, ^25^ ^]^

In 2015 we could show that also the bis‐silylated compound [Ge_9_(Hyp)_2_]^2−^ is available.^[^ ^26^ ^]^ It is thereby isostructural to previously published [Ge_9_R_2_]^2−^ compounds, like, e.g., [Ge_9_(EPh_2_)_2_]^2−^ (E═Sb, Bi),^[^ ^27^ ^]^ showing an almost unprotected germanium cluster core. This cluster exhibits a variety of different coordination types, as evidenced by its reaction with LnI_2_ (Ln═Yb, Eu, Sm), wherein the lanthanides show η^1^‐ η^2^‐ and η^3^ ‐coordination.^[^ ^28^ ^]^

In a direct comparison with the triple silylated cluster, the double silylated cluster was reacted with stabilised coinage metal halides. Fässler et al. used N‐heterocyclic carbenes for stabilisation. They were thus able to isolate the following clusters (NHC^Dipp^M)_2_[η^3^‐ Ge_9_(Hyp)_2_] (M═Cu, Ag, Au)^[^ ^29^ ^]^ and NHC^R^M[η^4^‐Ge_9_(Hyp)_2_] (R = Dipp, iPr, Mes; M═Cu, Ag, Au).^[^ ^30^ ^]^ Using *n*Bu_3_P as a stabiliser for AuCl, we were able to obtain the dimerised compound [(*n*Bu_3_PAu)_2_Ge_9_(Hyp)_2_]_2_.^[^ ^31^ ^]^ In this compound two different gold atoms are present, one in a terminal position and η^3^ bound to one Ge_9_ unit while the other is a bridging gold atom also η^3^ bound to one Ge_9_ and η^1^ bound to the second Ge_9_ unit.

In this study, we aim to expand upon this reactivity research by investigating the reaction between [Ge_9_(Hyp)_2_]^2−^ and phosphine‐stabilised silver chlorides, giving access to the unusual intermetalloid cluster Ag_12_[Ge_9_(Hyp)_2_]_6_ (**1**). (Figure 1) **1** is obtained from the reaction of K_2_[Ge_9_(Hyp)_2_] with (R_3_P)AgCl (R = Me, Et, Pr) at −78 °C in THF. Extraction with pentane and storage at 50 °C yields black diamond shaped crystals. The crystals formed over a period of three days in the oven in an overall yield of 19%. Compound **1** crystallizes in the trigonal crystal system, in the space group *R3*, where its structure can be refined as an inversion twin. The crystals are insoluble in all common organic solvents, hindering solution‐based characterization; NMR, ESI‐MS etc. However, further characterization was performed using XRD, EDX, XPS, and solid‐state NMR. The purity of the crystals was also verified using TGA and subsequent analysis of the residue by powder XRD. TGA also revealed that the crystals appear to be stable up to 220 °C.

**Figure 1:**
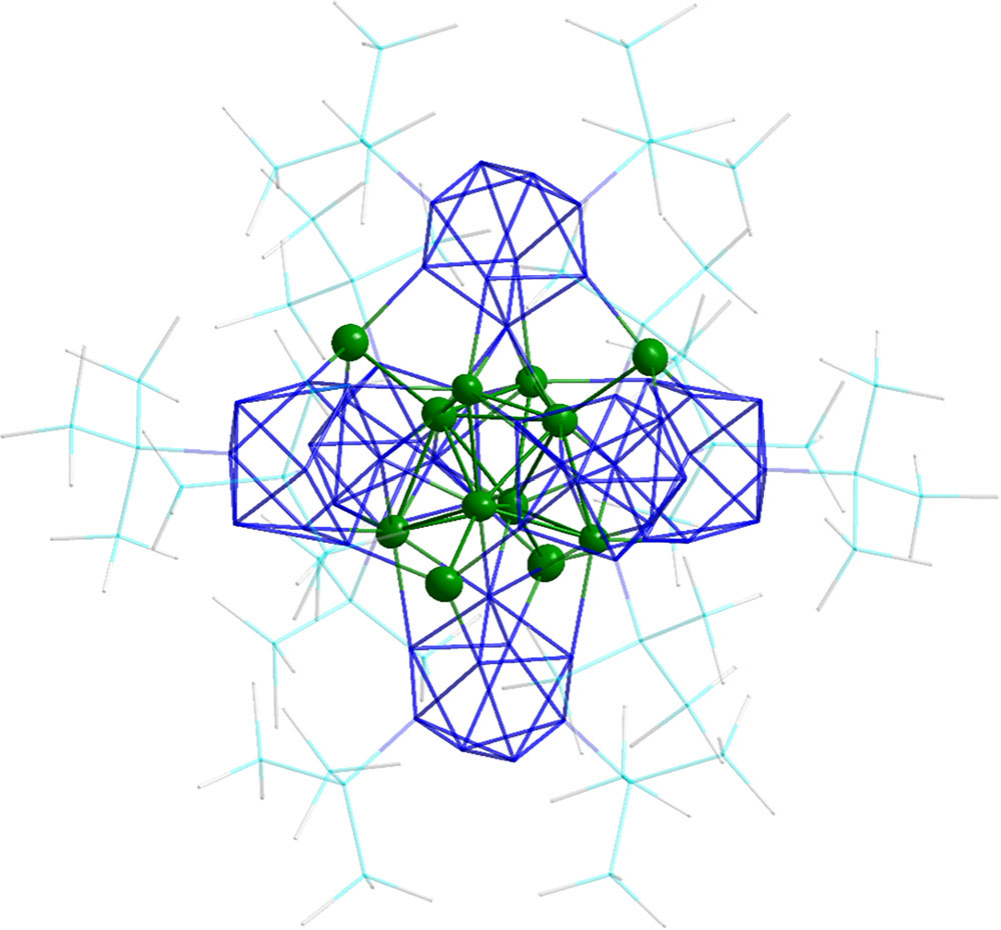
Molecular structure of **1**: Only the silver atoms (displayed in green) are shown as a thermal ellipsoid representation with 50% probability. Ge (blue), Si (turquoise) and C (gray) are shown as wires/sticks. H atoms are omitted for clarity. (Selected bond lengths and angles are shown in the Table S2).

Derived from the formula and the known compounds from literature, **1** might be seen as a hexamer of the [Ag_2_Ge_9_(Hyp)_2_] base unit. However, the molecular structure leads to a different interpretation that **1** is better described as an intermetalloid cluster exhibiting an Ag_12_ cluster core stabilized by six Ge_9_(Hyp)_2_ ligands (Figure 1). The Ag_12_ core can be described in two ways: first, as a tetra face and corner capped tetrahedron (Figure 2, left); or second, as three tetrahedral shells (Figure 2, right).

**Figure 2:**
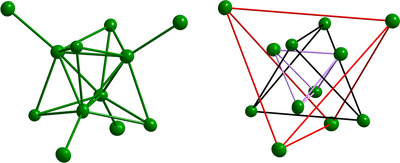
Two different viewpoints of the Ag_12_ core: Tetra face and corner capped tetrahedron (left) and three tetrahedron shells with the middle one rotated by 90° (inner tetrahedron: purple; middle tetrahedron: black; outer tetrahedron: red).

The silver–silver distances regarding the inner tetrahedron range from 2.968 to 2.982 Å, while the distances to the face capping silver atoms range from 2.982 to 3.126 Å. The distances for the corner capping silver atoms are with 2.797 to 2.811 Å much shorter, already indicating that different kinds of silver atoms are present (vide infra). The distances between the silver atoms in the Ag_12_ core are comparable to known distances in silver cluster compounds.^[^ ^32^, ^33^, ^34^ ^]^ Based on these distances, one might assume that the Ag_12_ core consists of two types of silver atoms. First, the eight silver atoms forming the face‐capped tetrahedron with many Ag─Ag contacts and second, the four silver atoms at the outer rim of the molecule, exhibiting mostly Ag─Ge contacts. The distances of the eight inner atoms are slightly longer than in elemental silver, while the distances from the inner tetrahedron to the four corner capping silver atoms are slightly shorter. The arrangement of the core's inner eight silver atoms of a tetra face capped tetrahedron is already known as substructure in metalloid silver cluster compounds. For example within [Ag_78_(DPPP)_6_(SR)_42_] (DPPP = 1,3‐bis(diphenyphosphino)propane; SR = SPhCF_3_).^[^ ^35^ ^]^ This arrangement is also found within intermetalloid silver‐copper clusters like Ag_20_Cu_10_(Dppm)_2_(SAdm)_14_Cl_8_ (Dppm = Bis(diphenylphosphino)methan; Adm = amantadinemercaptan).^[^ ^36^ ^]^ The exact structure is also know from an Ag_8_ cluster cation of Liu et al.^[^ ^37^ ^]^ However, this Ag_8_ Cluster shows some major differences, firstly it exhibits a hydride in the centre of the inner tetrahedron and secondly it is stabilised by a S_12_ icosahedron of six dtp ligands [dtp = S_2_P(OEt)_2_]. In case of **1** the Ag_12_ core has no inner hydride, and it is stabilised by six [Ge_9_(Hyp)_2_] units arranged in an octahedral fashion (Figure 3). **1** also fits to a family of lead clusters with precious metal cores, consisting of Au@Pb_11_ units which are arranged around a gold core. These compounds are synthesized from Pb_9_^4−^, the heavier homologue of Ge_9_^4−^ with Au(Mes)PPh_3_ undergoing thus substantial alteration of the group 14 precursor.^[^ ^38^ ^]^ In case of **1** the Ge_9_Hyp_2_ units undergo no changes, and the outer silver atoms are coordinated between the Ge_9_Hyp_2_ units rather than inside them.

**Figure 3:**
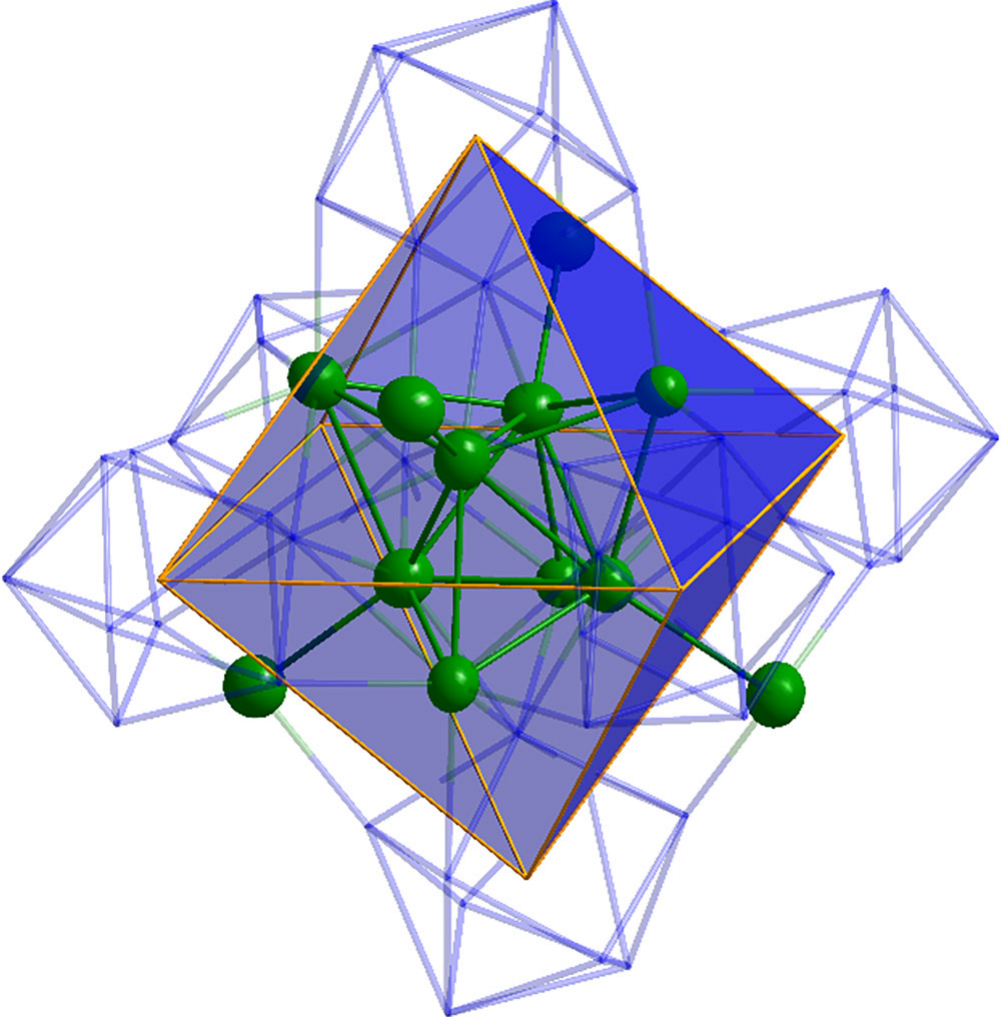
Left: The molecular structure of **1** without the Hyp‐ligands and the Ge atoms are shown in the wires and sticks model. Right: Molecular structure of **1** with Hyp‐ligands, Ge, Si and C atoms are shown in the wires and sticks model. Both: The octahedral arrangement of the Ge_9_ units is emphasized by polyhedral representation. The corners of the octahedron are placed in the centre of the Ge_9_ units.

The six Ge_9_ units are positioned over the six edges of the inner tetrahedron of the Ag_12_ silver core, resulting in an octahedral arrangement of the six Ge_9_(Hyp)_2_ units. The twelve Hyp ligands are thereby precisely aligned along the twelve edges of the constructed octahedron.

Each of the six Ge_9_ units can be described as a distorted face capped trigonal prism. Thereby, two of the capping germanium atoms (Ge1 and Ge3) are bound to a Hyp‐ligand, while the third atom (Ge9) aligns with the edge of the inner tetrahedron of the Ag_12_ core and exhibits an η^4^‐coordination with the Ag_12_ core (Figure 4). The contact distances of Ge9 to the silver atoms Ag2, Ag2´, Ag1, and Ag4 are 2.626, 2.618, 2.919, and 2.888 Å respectively. The germanium atoms Ge5 and Ge7 are η^1^‐coordinated to Ag4 and Ag1 with distances of 2.862 Å, 2.854 Å, while Ge6 and Ge8 are η^1^‐coordinated to the corner‐capping silver atoms Ag3 and Ag3´ with contact distances of 2.448 and 2.583 Å respectively. Known Ge‐Ag distances in molecular compounds range from 2.402 to 2.448 Å.^[^ ^39^, ^40^, ^41^ ^]^ For direct comparison, the intermetalloid clusters of our and Fässler´s working group are important, where the Ag‐Ge distances range from 2.734 to 2.764 Å.^[^ ^18^, ^42^ ^]^ The distances between the inner eight silver atoms to Ge_9_ are thus comparable to the previously known distances between silver and Ge_9_, while the distances between the outer silver atoms and Ge_9_ are significantly shorter and more comparable to the distances in molecular compounds.

**Figure 4:**
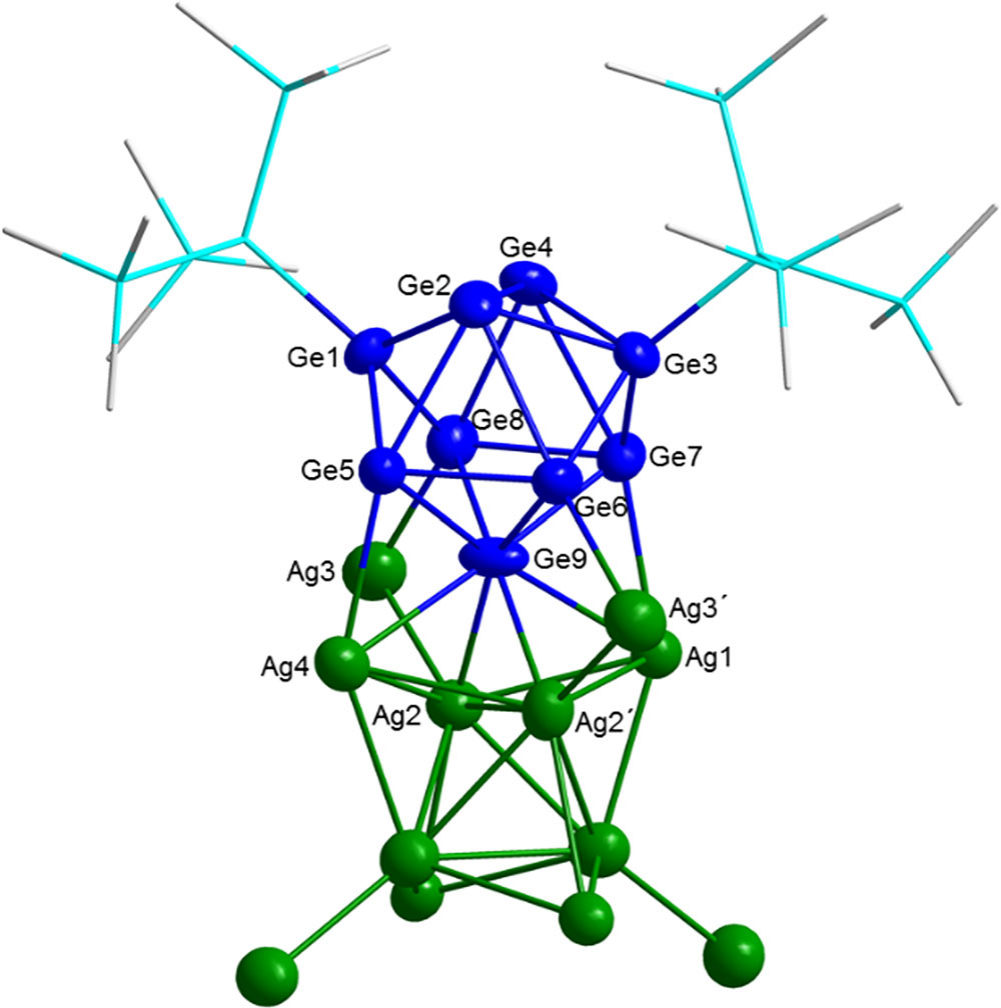
Coordination of one Ge_9_(Hyp)_2_ unit to the Ag_12_ core. Si and C atoms are shown as wires/sticks and H atoms are omitted for clarity.

To investigate the presence of different kinds of silver atoms within **1** in more detail, we performed X‐ray photoelectron spectroscopy (XPS) studies, (Figure 5) allowing further insight on the oxidation states of the Ag and the Ge atoms present in compound **1**.

**Figure 5:**
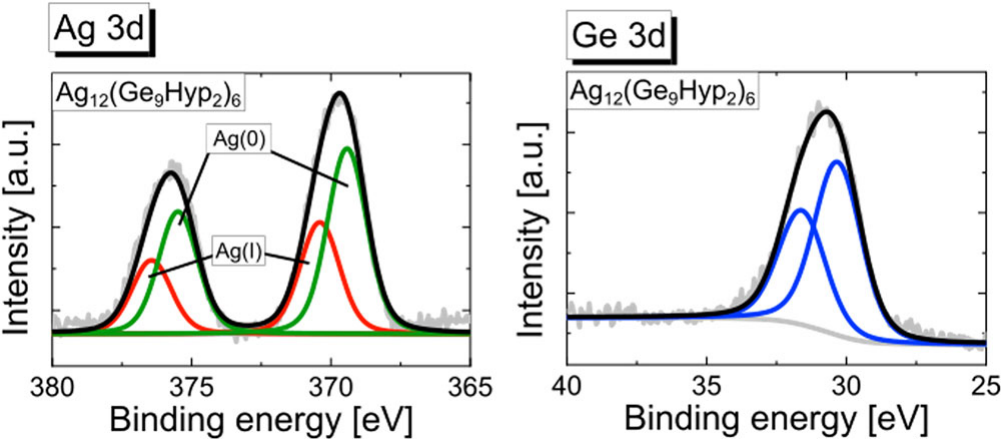
XPS spectra of Ag_12_[Ge_9_(Hyp)_2_]_6_: a) Binding Energy of Ag 3d, fit for the measured energies in black, the fit for the Ag(I) component in red and the fit for the Ag(0) component in green; b) Binding Energy of Ge 3d fit for the measured energies in black fit for spin‐orbit splitting into Ge 3d_5/2_ and Ge 3d _3/2_ in blue.

The XPS spectra of Ag 3d (Figure 5a) were fitted using two distinct components (green and red lines; fit parameters are listed in Table S6). Each component exhibits a characteristic spin‐orbit splitting into Ag 3d_5/2_ and Ag 3d_3/2_ peaks. The lower binding energy component (green line) exhibits an Ag 3d_5/2_ peak, which lies between the typical binding energies observed for metallic Ag^0^ and Ag^I^. In contrast, the higher binding energy component (red line) is in good agreement with the measured value for Ag^I^ in (Et_3_P)AgCl (Figure S3).

The relative intensities of the two compounds suggest an approximate ratio of 1:2. Based on this, we propose that the lower binding energy component corresponds to the eight Ag atoms less coordinated to germanium, while the higher binding energy component can be assigned to the four Ag atoms that are more directly bound to germanium. The Ge 3d core‐level spectrum (Figure 5b) shows the spin‐orbit doublet with Ge 3d_5/2_ and Ge 3d_3/2_ peaks located at binding energies of 30.3 and 31.6 eV, respectively. Consequently, germanium atoms show the expected oxidation state between 0 and + I.

Based on the structure, the silver distances and the XPS results, we assume that the four silver atoms of the inner tetrahedron, along with the four face‐capping silver atoms, are in an oxidation state lower than one. Meanwhile, the four outer silver atoms probably remain in an oxidation state of one. To further underline this, we performed quantum chemical calculations on the geometry‐optimized isostructural model compound Ag_12_[Ge_9_(SiH_3_)_2_]_6_ **1′**. The population analysis based on occupation numbers, at the silver atoms, gives significant differences in the calculated charge values, underlining the 2:1 ratio found in the XPS studies. For the four outer Ag atoms, an average partial charge at +0.3 was calculated. This corresponds to a classical Ag^+^ cationic description as found in [AgGe_18_(Hyp)_6_]^−^. For the other eight Ag atoms located in the centre of compound **1** negative values (−0.4 to −0.5) are calculated. These partial charges underline the experiments finding of electron rich Ag atoms, which are thus more like Ag atoms found in cluster compounds and can be formally described with an oxidation state of zero. This result directly shows that not only an oligomer of Ag_2_Ge_9_(Hyp)_2_ is present, but that an intermetalloid cluster has formed where electron density has been transferred from the [Ge_9_(Hyp)_2_]^2‒^ ligands to the silver cations of the cluster core.

In this work further investigations of the reactivity of K_2_Ge_9_(Hyp)_2_ are presented. Thereby the reaction with a phosphine stabilised silver chloride, gives the intermetalloid cluster Ag_12_[Ge_9_(Hyp)_2_]_6_ (**1**) from a pentane extract at 50 °C. The cluster shows an interesting face and corner covered tetrahedral Ag_12_ core surrounded by six [Ge_9_(Hyp)_2_] units in an octahedral pattern. Due to the structure and contact distances within the silver core, two different types of silver atoms might be assumed. XPS measurements and quantum chemical calculations support this interpretation, showing that eight silver atoms are best described as Ag(0) while four silver atoms are described as silver cations Ag^+^. Consequently, a redox chemistry has taken place during the formation of the intermetalloid cluster compound **1**. If this redox chemistry might be further expanded to larger metalloid or intermetalloid silver cluster compounds must be checked by ongoing investigations in this reaction system.

The authors thank the Deutsche Forschungsgemeinschaft (DFG) for financial support. The authors acknowledge support by the state of Baden‐Württemberg through bwHPC and the German Research Foundation (DFG) through grant no INST 40/575–1 FUGG (JUSTUS 2 cluster).

Open access funding enabled and organized by Projekt DEAL.

## Conflict of Interests

The authors declare no conflict of interest.

## Supporting Information

The authors have cited additional references within the Supporting Information.^[^ ^43^, ^44^, ^45^, ^46^, ^47^, ^48^, ^49^, ^50^, ^51^, ^52^, ^53^, ^54^, ^55^ ^]^

## Supporting information



Supporting Information

## Data Availability

The data that support the findings of this study are available from the corresponding author upon reasonable request.
