# Supplementary material for: Ag12[Ge9(Hyp)2]6 An Intermetalloid Cluster with Bis‐Silylated Ge9 Units
Source: Angew Chem Int Ed Engl. 2025 Dec 4;65(4):e21991. doi: 10.1002/anie.202521991 (PMC12828458; doi:10.1002/anie.202521991)
Supplement: Supplementary file 1 — Supporting Information [file ANIE-65-e21991-s001.pdf]

Supporting Information

$Ag_{12}\{Ge_9Hyp_2\}_6$: An Intermetalloid Cluster with bis-Silylated Ge_9-Units

Kevin Wörn, Claudio Schrenk, Eric Juratti and Andreas Schnepf*

Chemistry Department , University of Tuebingen, Germany

1. General Methods

All handling of materials was performed under exclusion of air and moisture, either by using dried Schlenk type flasks or an argon filled glovebox. Solvents were refluxed over drying agents (THF, toluene and Benzene over sodium, Pentane over CaH_2 and Hexane over a Na/K alloy) for one hour, then distilled and degassed before storage over a Na/K alloy.

$(R_3P)AgCl$ (R = Me, Et, Pr)

AgCl (1 eq.) was suspended in toluene then R_3P (1 eq.) is added to the solution, which is then stirred for 12 hours. The solution is then filtered, and the toluene is slowly removed under reduced pressure. $(R_3P)AgCl$ is obtained as colourless crystals with a yield of 80-90%.

$K_2Ge_9Hyp_2$ (Hyp = $Si(SiMe_3)_3$)

K_4Ge_9 (2g, approximately 1.6 g of K_4Ge_9, 0.2 mmol, 1 eq.) is mixed with a solution of HypBr (1.2 g, 0.37 mmol, 1.9 eq.) in acetonitrile (20 mL). The reaction mixture is stirred for five days and then filtered off from the remaining solid. The acetonitrile is removed under reduced pressure and the residue is washed twice with pentane (10 mL) and once with toluene (5 mL). $K_2Ge_9Hyp_2$ is thus obtained as a golden brown powder in a yield of 80% with respect to the applied HypBr.

$Ag_{12}[Ge_9(Hyp)_2]_6$ (**1**)

$K_2Ge_9Hyp_2$ (123 mg, 0.1 mmol, 1 eq.) and $(Et_3P)AgCl$ (52 mg, 0.2 mmol, 2 eq.) are put together and dissolved in THF (20 mL). The brown solution is then stirred for an hour at –78°C. Afterwards the THF is removed under reduced pressure. The dried reaction mixture is then extracted with hexane (15 ml). The extract is then stored at –30 °C for 12 hours, there some amorphous solids precipitate. The solution then is filtrated in a new flask which is then again stored at –30 °C. This process is repeated till no solids precipitate anymore then the solution is stored at 50 °C where after two days, black diamond shaped crystals can be obtained (25 mg, 0,0031 mmol, 19% yield). The crystals are after crystallisation not soluble in any common solvent. Characterisation was done via x-ray crystal structure analysis, EDX, XPS and solid-state NMR (TableS1-S2, TableS3-S5, TableS6, FigureS4). The purity of the compound was further determent through TGA with subsequent analysis of the residue

through powder XRD. The synthetic procedure is the same for the reactions of $(Me_3P)AgCl$ and $(Pr_3P)AgCl$ with $K_2Ge_9Hyp_2$.

2. Crystallographic section

Crystals of compound **1** were mounted on the diffractometer at 200 K. The data were collected on a Rigaku XtaLAB Synergy-S X-ray diffractometer for single crystal X-ray diffractometer equipped with a PhotonJet-S microfocus sealed tube for monochromated MoKα radiation (λ = 0.71073 Å) and equipped with an Oxford Cryosystems cryostat. A semiempirical absorption correction using spherical harmonics was applied using the SCALE3 ABSPACK algorithm. The structure was solved by direct methods and refined against F^2 for all observed reflections. Programs used: SHELXT and SHELXL[43-44] within the Olex2 program package.[45]

The primary structure solution hints to the centrosymmetric space group $R\overline{3}$. This solution fits perfectly with the octahedral arrangements of the $Ge_9[Hyp_2]$ units in the outer shell of the molecule. In this case only one $Ge_9[Hyp_2]$ unit is found in the asymmetrical unit. Since the convoluted tetrahedral arrangement of the Ag atoms excludes an inversion center, we could not split the silver positions shown as symmetry equivalents, but could not be equivalent at all. Therefore, we try to refine the structure in the chiral space group R3, where now we were able to clearly identify the Ag positions by refining with a disorder model of 57:43 ratio. The calculated Flack parameter of the chiral refinement is with 0.45 in line with the disorder model, therefore an inversion twin refinement is done using TWIN -1 0 0 0 -1 0 0 0 -1 and a scaling factor of 0.45.

Due to additional disordering of the Hyp substituent, which is well-known for that type, we fixed the substituent's structure with hard and soft restraints, like SADI, SIMU, RIGU and DFIX. To get reliable results, the disordered Ag atoms' thermal ellipsoids were last treated with SIMU during the refinement.

Co-crystallized solvent molecules could not be refined properly, therefore SQUEEZE was used to identify and model 18 hexane molecules in the unit cell.

The H atom positions in all compounds were refined using a riding model. The supplementary crystallographic data (for CCDC numbers, see Table S1) can be obtained online free of charge at www.ccdc.cam.ac.uk/conts/retrieving.html or from Cambridge Crystallographic Data Centre, 12 Union Road, Cambridge CB21EZ; Fax: (+44)1223-336-033; or deposit@ccdc.cam.ac.uk.

Table S1: Data of the crystal structure determination of compound **1**

Empirical formula	$C_{108}H_{324}Ag_{12}Ge_{54}Si_{48}$
Formula weight	8186.27
Temperature/K	200.0(3)
Crystal system	trigonal
Space group	R3
a/Å	23.4351(3)
b/Å	23.4351(3)
c/Å	50.7183(6)
α/°	90
β/°	90
γ/°	120
Volume/Å^3	24122.9(7)
Z	3
ρ_{calc}g/cm^3	1.691
μ/mm^{-1}	5.870
F(000)	11808.0
Crystal size/mm^3	0.194 × 0.099 × 0.086
Radiation	Mo Kα (λ = 0.71073)
2Θ range for data collection/°	3.788 to 52.742
Index ranges	-29 ≤ h ≤ 29, -29 ≤ k ≤ 28, -63 ≤ l ≤ 63
Reflections collected	117345
Independent reflections	21615 [R_{int} = 0.0465, R_{sigma} = 0.0401]
Data/restraints/parameters	21615/405/780
Goodness-of-fit on F^2	1.028
Final R indexes [I>=2σ (I)]	R_1 = 0.0526, wR_2 = 0.1366
Final R indexes [all data]	R_1 = 0.0798, wR_2 = 0.1514
Largest diff. peak/hole / e Å^{-3}	1.69/-0.71
Flack parameter	0.455(19)
CCDC number	2493892

Table S2 Selected Bond Lengths for **1**

Ag1	Ag2	2.950(5)	Ge3	Ge6	2.506(3)	Ag5	Ge19^{2}	2.682(3)	Ge17	Ge18	2.527(3)
Ag1	Ag2^{1}	2.950(5)	Ge3	Ge7	2.502(3)	Ag5	Ge19	2.682(3)	Ge17	Ge19	2.604(4)
Ag1	Ag2^{2}	2.950(5)	Ge3	Si2	2.361(6)	Ag5	Ge19^{1}	2.682(3)	Ge18	Ge19	2.598(4)
Ag1	Ge7^{1}	2.854(3)	Ge4	Ge7	2.825(3)	Ag6	Ge15^{2}	2.523(2)	Ag10	Ag12	2.962(5)
Ag1	Ge7^{2}	2.854(3)	Ge4	Ge8	2.727(3)	Ag6	Ge15^{1}	2.523(2)	Ag10	Ge9	2.627(3)
Ag1	Ge7	2.854(3)	Ge5	Ge6	2.527(3)	Ag6	Ge15	2.523(2)	Ag10	Ge19	2.610(4)
Ag1	Ge9^{2}	2.919(3)	Ge5	Ge9	2.608(4)	Ag7	Ag8	2.850(6)	Ag10	Ge19^{1}	2.633(4)
Ag1	Ge9^{1}	2.919(3)	Ge6	Ge9	2.596(4)	Ag7	Ge7^{1}	2.532(2)	Ag11	Ge5	2.516(4)
Ag1	Ge9	2.919(3)	Ge7	Ge8	2.529(3)	Ag7	Ge7	2.531(2)	Ag11	Ge16	2.469(4)
Ag2	Ag2^{1}	2.969(6)	Ge7	Ge9	2.575(3)	Ag7	Ge7^{2}	2.532(2)	Ag11	Ge18^{1}	2.586(3)
Ag2	Ag2^{2}	2.969(6)	Ge8	Ge9	2.633(3)	Ag8	Ag9^{1}	2.996(3)	Ag12	Ge15^{1}	2.823(3)
Ag2	Ag3	2.811(5)	Ge11	Ge12	2.551(3)	Ag8	Ag9	2.996(3)	Ag12	Ge15^{2}	2.823(3)
Ag2	Ag4	2.982(4)	Ge11	Ge14	2.560(3)	Ag8	Ag9^{2}	2.996(3)	Ag12	Ge15	2.823(3)
Ag2	Ag4^{2}	3.126(5)	Ge11	Ge15	2.502(3)	Ag8	Ag10	2.961(5)	Ag12	Ge19^{2}	2.966(3)
Ag2	Ag5	2.982(6)	Ge11	Ge18	2.505(3)	Ag8	Ag10^{1}	2.961(5)	Ag12	Ge19^{1}	2.966(3)
Ag2	Ge9	2.626(4)	Ge11	Si3	2.418(5)	Ag8	Ag10^{2}	2.961(5)	Ag12	Ge19	2.966(3)
Ag2	Ge9^{2}	2.618(4)	Ge12	Ge13	2.584(3)	Ag8	Ge9	2.658(2)	Ge1	Ge2	2.580(3)
Ag2	Ge19	2.598(4)	Ge12	Ge14	2.896(4)	Ag8	Ge9^{2}	2.658(2)	Ge1	Ge4	2.571(3)
Ag3	Ge6	2.583(4)	Ge12	Ge15	2.840(3)	Ag8	Ge9^{1}	2.658(2)	Ge1	Ge5	2.513(3)
Ag3	Ge8^{2}	2.448(4)	Ge12	Ge16	2.715(3)	Ag9	Ag10	3.110(4)	Ge1	Ge8	2.533(3)
Ag3	Ge17	2.480(4)	Ge13	Ge14	2.578(3)	Ag9	Ag10^{2}	2.984(4)	Ge1	Si1	2.397(6)
Ag4	Ag5	3.032(4)	Ge13	Ge16	2.532(3)	Ag9	Ge6	2.680(3)	Ge2	Ge3	2.573(3)
Ag4	Ge5	2.862(4)	Ge13	Ge17	2.518(3)	Ag9	Ge8^{2}	2.948(4)	Ge2	Ge4	2.872(4)
Ag4	Ge9	2.888(4)	Ge13	Si4	2.399(6)	Ag9	Ge9	2.996(4)	Ge2	Ge5	2.730(3)
Ag4	Ge16	2.948(4)	Ge14	Ge17	2.745(3)	Ag9	Ge9^{2}	2.777(4)	Ge2	Ge6	2.807(3)
Ag4	Ge18^{1}	2.689(4)	Ge14	Ge18	2.783(3)	Ag9	Ge17	2.873(4)	Ge3	Ge4	2.560(3)
Ag4	Ge19	2.773(4)	Ge15	Ge16	2.519(3)	Ag9	Ge19	2.876(4)			
Ag4	Ge19^{1}	3.046(4)	Ge15	Ge19	2.581(3)	Ag10	Ag10^{1}	2.932(5)			
Ag5	Ag6	2.796(8)	Ge16	Ge19	2.602(4)	Ag10	Ag10^{2}	2.932(5)			

3. EDX/SEM studies

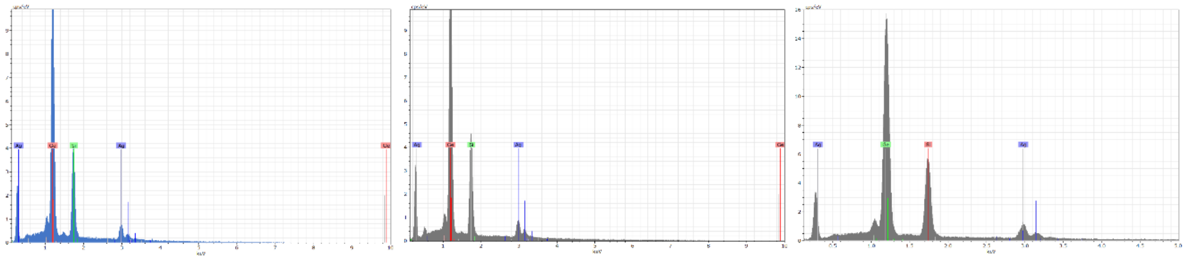

Figure S1: EDX spectra of the points 5 (left) and 6 (middle) and 15 (right)

Table S3: Point 5

Element	Norm Wt. %	Narm Wt. % calculated	Norm At%	Norm At% calculated	Error (Wt% 3 σ)
Germanium	58,87609	59.74336	46,61052	47.36842	2,36170
Silizium	20,79137	20.53869	42,55419	42.10526	0,67008
Silber	20,33254	19.71795	10,83529	10.52632	0,57743

Table S4: Point 6

Element	Norm Wt. %	Narm Wt. % calculated	Norm At%	Norm At% calculated	Error (Wt% 3 σ)
Germanium	59,16269	59.74336	46,4883	47.36842	2,41410
Silizium	21,23845	20.53869	43,14524	42.10526	0,69373
Silber	19,59886	19.71795	10,36646	10.52632	0,56009

Table S5: Point 15

Element	Norm Wt. %	Narm Wt. % calculated	Norm At%	Norm At% calculated	Error (Wt% 3 σ)
Germanium	58,87609	60,02681	47,73143	47.36842	2,73417
Silizium	20,79137	20,30415	41,74057	42.10526	0,73677
Silber	20,33254	19,66903	10,52800	10.52632	0,61538

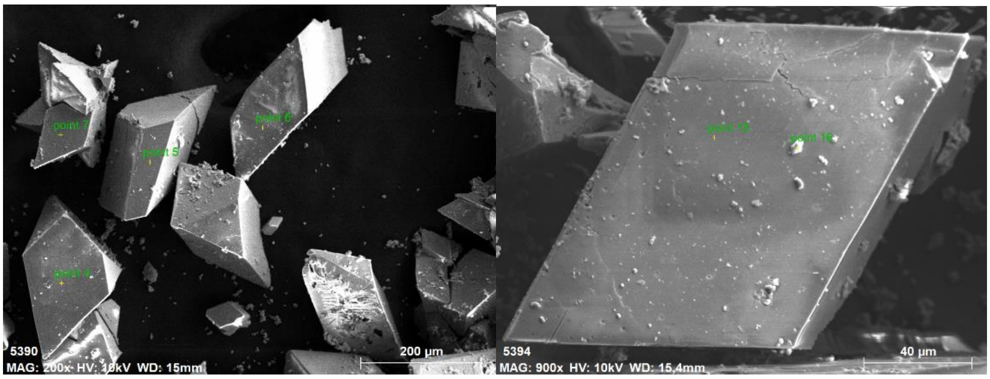

Figure S2: SEM Image of the crystals of compund **1**

4. XPS Measurement

XPS measurements were performed at a home laboratory setup under ultrahigh vacuum (UHV) conditions (base pressure 6 x 10^{-10} mbar) equipped with an Al Kα radiation source (XR 50, SPECS GmbH) and a hemispherical analyzer (Phoibos 150, SPECS). Ar-ion sputtered foils of Au, Ag and Cu (Goodfellow Cambridge Ltd.) were used for energy calibration at the binding energies of Au $4f_{7/2}$ (84.00 eV), Ag $3d_{5/2}$ (368.21 eV) and Cu $2p_{3/2}$ (932.63 eV).

All XPS peak fits were performed with Unifit Software (Unifit Scientific Software GmbH) using Voigt profiles as a convolution of Gaussian and Lorentzian line shapes, and with Shirley backgrounds.

Table S6: Fit parameters for the XPS experiments depicted in Figure 5a and 5b

**5a**	Position (eV)	Gaussian width (eV)	Lorentzian width (eV)	Rel. Area (%)
Ag(I) $3d_{5/2}$	370.4	1.5	0.4	22.8
Ag(I) $3d_{3/2}$	376.4	1.5	0.4	14.9
Ag(0) $3d_{5/2}$	369.4	1.5	0.4	37.6
Ag(0) $3d_{3/2}$	375.5	1.5	0.4	24.7
**5b**	Position (eV)	Gaussian width (eV)	Lorentzian width (eV)	Rel. Area (%)
Ge $3d_{5/2}$	30.3	1.7	0.5	60.2
Ge $3d_{3/2}$	31.6	1.7	0.5	39.8

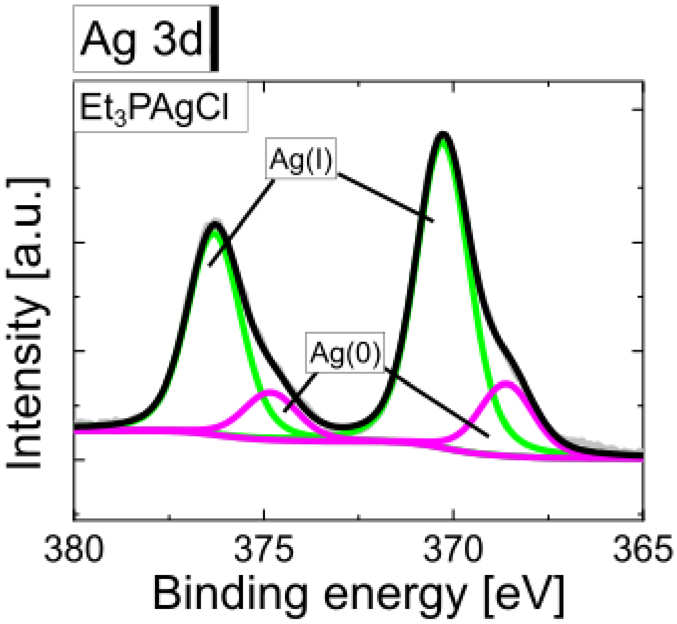

Figure S3: XP spectra of Et_3PAgCl: Binding Energy of Ag 3d, fit for the measured energies in black, the fit for the Ag(I) component in green and the fit for the Ag(0) component in pink.

Table S7: Fit parameters for the XPS experiments depicted in Figure S3.

**5a**	Position (eV)	Gaussian width (eV)	Lorentzian width (eV)	Rel. Area (%)
Ag(I) $3d_{5/2}$	370.3	1.4	0.5	48.7
Ag(I) $3d_{3/2}$	376.3	1.4	0.5	31.9
Ag(0) $3d_{5/2}$	368.6	1.4	0.5	11.7
Ag(0) $3d_{3/2}$	374.8	1.4	0.5	7.7

5. Solid State NMR

The ^{29}Si cross-polarization magic-angle-spinning (CP/MAS) spectrum was obtained on a 300 MHz Bruker Avance III HD NMR spectrometer equipped with a wide-bore magnet, a 4-mm-o.d. MAS probe head, operating at 300.13 MHz (^{1}H) and 59.63 MHz (^{29}Si).

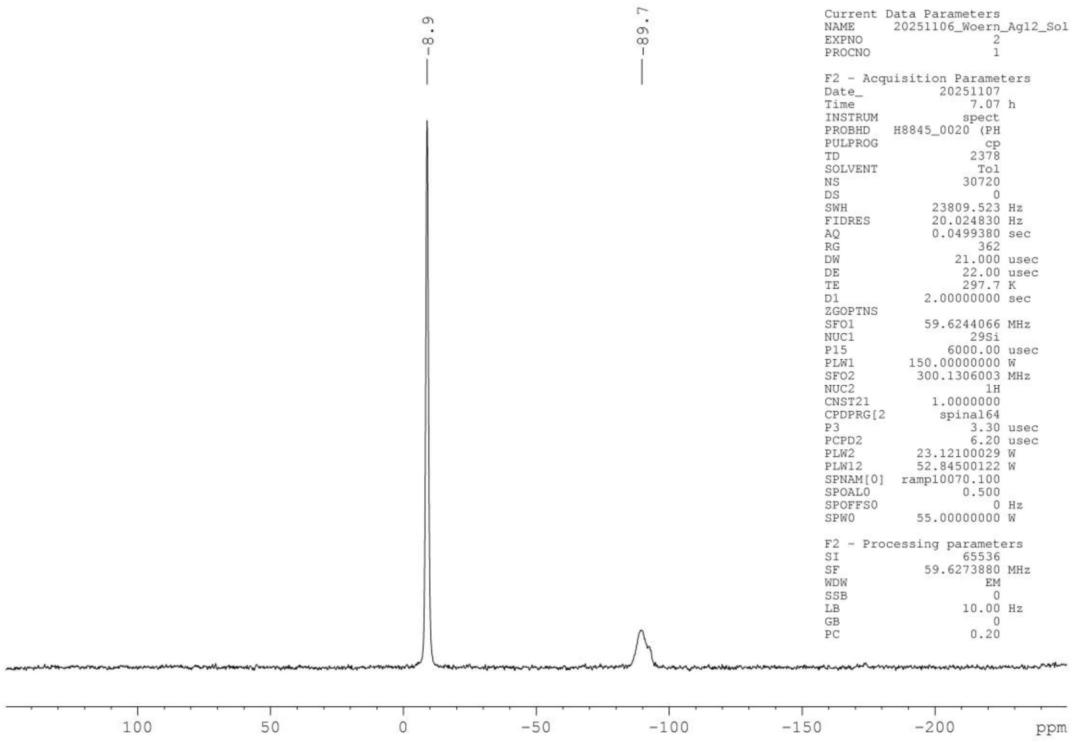

Figure S4: Solid state ^{29}Si-NMR of $Ag_{12}[Ge_9(Hyp)_2]_6$ (**1**) at 25 °C. Signal at -8.9 ppm can be assigned to the silicon atoms of the TMS groups and the signal at -89.7 ppm to the central silicon atom of the Hyp groups.

6. TGA and XRPD

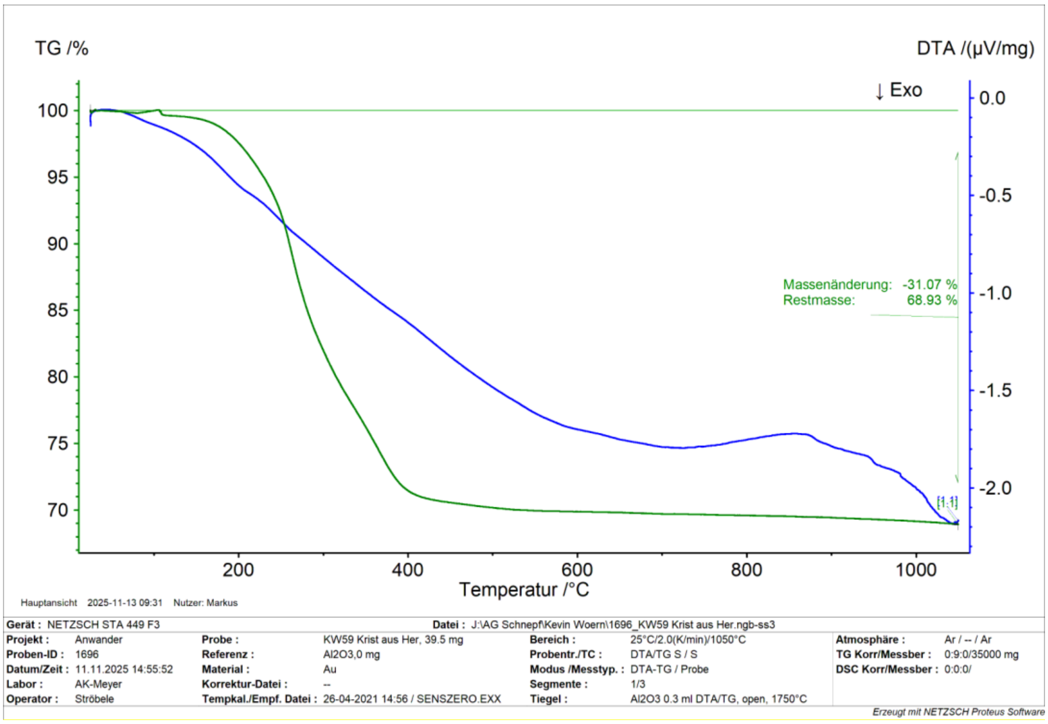

Figure S5: Thermogravimetric analysis of 39.5 mg of compound **1**, a mass loss of -11.941 mg is observable (30.23%). The decomposition starts at around 200 °C and ends at around 390 °C. The Measurement was performed under inert conditions with an Argon flow rate of 240.3 mL/min.

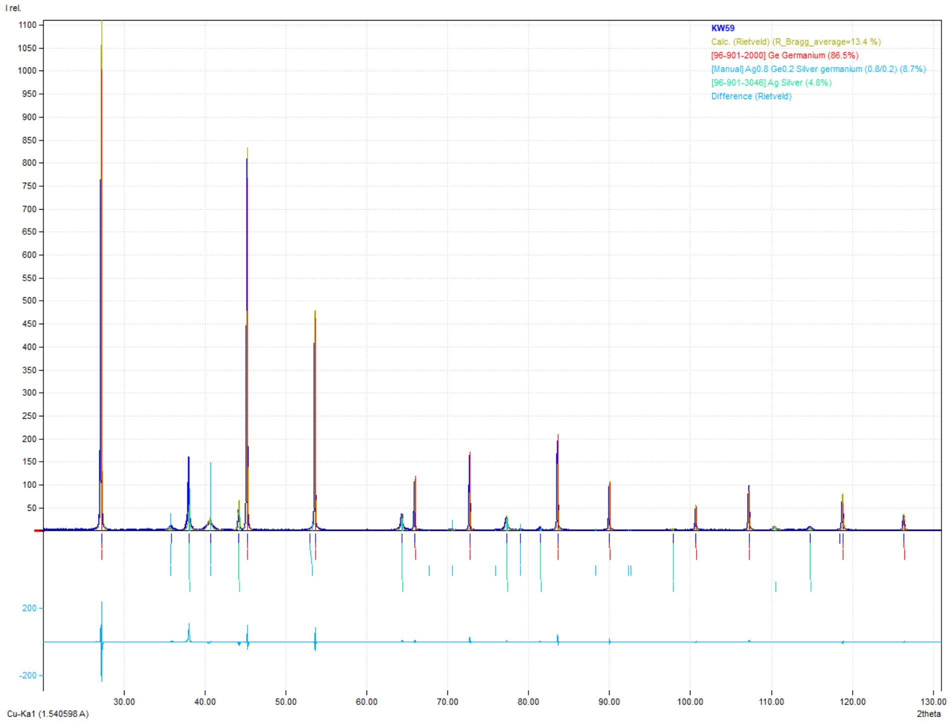

Figure S6: X-ray powder diffraction pattern of the residue from the thermogravimetric analysis. It shows signals for germanium, silver and an alloy of (Ag0.8/Ge0.2). The weight ratio is not accurate, as the silver is not in powder form after the TGA but in small spheres and therefore cannot be completely detected.

7. Quantum chemical calculations

Quantum-chemical calculations were carried out with the RI-DFT version[46] of the TURBOMOLE[47] program package by employing the BP86-functional.[48-49] The basis sets were of SVP quality.[50] The TmoleX client[51] was used as graphical user interface and for illustrating the molecular orbitals. PABOON[52-53] was used to calculate the partial charges.

7.1 Model compound $Ag_{12}(Ge_9(SiH_3)_2)_6$

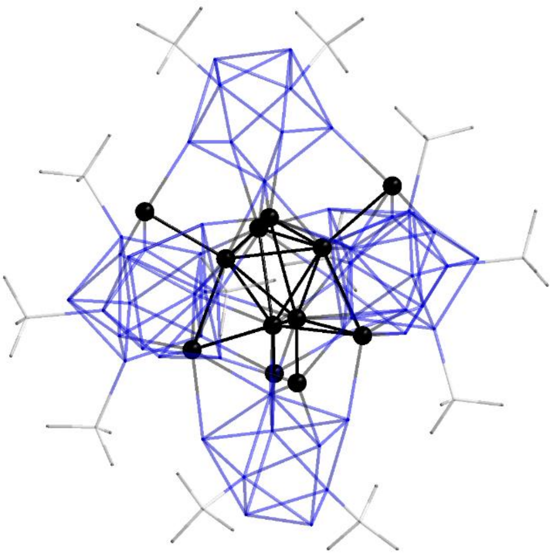

Figure S7: Geometry optimized structure.

Point group: C_1

Energy: -117421.71428597558 Hartree

HOMO-LUMO-gap: 1.057 eV

Table S8: Atomic coordinates and partial charges:

Atom	coordinates			partial charge
Ag	-0.197619	13.397.354	39.152.143	-0.1257
Ag	-0.257514	11.854.933	41.649.938	-0.3256
Ag	1.644.252	14.221.055	41.354.631	-0.5405
Ag	-1.246.331	14.775.544	41.631.911	-0.3990
Ge	0.559331	15.851.606	38.562.177	0.2254
Ge	-2.468.693	12.408.832	37.848.470	0.0641
Ge	1.708.259	11.758.413	38.256.374	0.0543
Ge	-2.577.579	12.707.189	40.554.621	0.0834
Ge	0.448255	16.790.459	41.044.579	0.1965
Ge	2.435.343	11.677.557	40.785.507	0.1209

Ag	-1.266.926	9.309.250	40.657.586	0.4118
Ag	2.051.586	11.303.535	43.550.941	-0.5486
Ag	-2.779.650	12.825.686	43.453.894	-0.4185
Ag	0.126441	13.566.869	44.078.028	-0.4607
Ge	-0.684754	10.966.616	44.180.286	0.1730
Ge	1.237.211	9.387.040	39.966.068	0.1157
Ge	-3.226.414	10.457.196	39.347.663	0.0326
Ge	-1.717.721	8.725.579	43.166.579	0.1014
Ge	3.066.150	9.231.813	41.809.164	0.0438
Ge	1.219.196	9.570.629	45.587.922	0.1151
Ge	4.355.304	12.366.745	44.511.016	0.2229
Ge	2.669.910	14.373.131	43.940.500	0.0050
Ag	0.102704	13.533.274	46.993.987	0.4432
Ge	-1.829.655	15.431.831	44.224.329	0.2528
Ge	-1.933.667	15.160.886	46.861.807	0.1750
Ge	2.552.187	14.311.247	46.584.827	0.1711
Ge	-0.504185	11.001.013	46.818.405	0.0456
Ge	4.854.667	10.284.587	40.265.339	-0.0805
Ge	3.730.634	8.023.522	39.436.229	0.1100
Ge	4.827.596	9.970.214	37.687.137	0.0993
Ge	4.172.987	12.368.162	38.792.549	-0.0689
Si	7.144.553	10.294.565	41.017.593	0.0454
Ge	2.331.995	9.384.993	37.632.947	-0.1539
Si	1.165.957	7.943.120	36.099.450	0.0322
Ge	-2.703.207	9.736.783	46.857.548	-0.1022
Ge	-0.609654	8.217.036	47.348.564	0.1039
Ge	-2.738.573	7.534.947	45.483.658	0.1018
Ge	-3.294.008	10.274.846	44.450.154	0.2339
Si	-4.183.665	9.858.774	48.750.300	0.0334
Ge	-0.187951	7.590.273	44.851.985	-0.0792
Si	0.612264	5.340.839	44.531.330	0.0370
Ag	4.400.737	14.330.617	40.500.777	0.2131
Ag	0.838684	16.535.602	43.744.702	-0.4834
Ge	3.035.928	16.580.469	40.080.420	0.2715
Ag	-2.857.616	17.021.661	40.727.065	0.3124
Ge	-4.163.871	14.800.637	40.051.512	0.1757
Ge	5.179.582	14.284.635	43.013.567	-0.0092
Ge	2.417.485	18.433.480	41.801.129	0.0637
Ge	2.864.409	16.646.839	45.575.170	-0.0634
Ge	-1.236.845	18.043.440	44.699.286	0.2524
Ge	3.064.927	19.359.720	39.259.426	0.1047
Ge	2.386.783	17.349.778	37.689.604	-0.1134
Ge	-1.044.145	17.828.572	39.021.963	0.0314
Ge	-3.275.143	17.466.489	43.273.445	0.0771
Ge	-5.001.108	13.042.425	41.734.850	0.1942
Ge	-3.951.567	14.303.168	45.557.922	0.0790
Ge	-6.533.520	13.328.024	39.318.434	0.0765

Ge	-4.536.829	13.877.689	37.727.042	-0.1002
Ge	5.183.051	16.329.839	44.558.927	-0.0733
Ge	6.652.964	14.285.854	45.337.844	0.1073
Ge	0.618120	19.480.396	40.219.560	-0.0804
Ge	5.012.901	15.839.473	47.134.582	0.1168
Ge	-2.099.370	17.680.756	47.050.484	-0.1095
Ge	-3.983.360	18.773.907	45.640.338	0.1060
Ge	0.745125	19.323.200	37.615.974	0.0926
Si	4.123.230	16.770.293	36.127.663	0.0294
Ge	4.820.346	13.240.335	46.837.302	-0.0969
Ge	-5.026.162	16.494.013	44.844.214	-0.0823
Ge	-5.556.225	11.080.583	40.190.911	-0.0978
Ge	-4.416.760	16.487.853	47.378.391	0.1072
Ge	-5.185.119	11.368.943	37.594.428	0.0973
Si	-5.125.210	15.245.533	35.840.242	0.0386
Si	6.729.081	18.140.095	44.157.033	0.0417
Si	-0.326342	21.586.609	40.913.412	0.0400
Si	-1.338.336	18.862.268	49.002.268	0.0346
Si	5.377.291	11.893.056	48.746.263	0.0359
Si	-7.378.259	16.815.595	44.430.271	0.0392
Si	-7.270.690	9.434.247	40.589.218	0.0448
H	-5.167.672	8.717.136	48.717.795	-0.0561
H	-3.382.258	9.788.079	50.024.544	-0.0554
H	-4.941.368	11.161.094	48.716.981	-0.0565
H	8.033.957	10.914.916	39.970.950	-0.0542
H	7.246.458	11.093.591	42.291.075	-0.0533
H	7.595.677	8.881.070	41.285.128	-0.0559
H	6.806.984	11.429.196	48.638.645	-0.0566
H	4.468.621	10.693.043	48.803.523	-0.0545
H	5.211.474	12.703.701	50.005.641	-0.0541
H	0.162611	18.770.660	49.097.281	-0.0558
H	-1.738.413	20.312.695	48.912.354	-0.0566
H	-1.948.914	18.253.909	50.238.229	-0.0557
H	-1.268.159	22.112.220	39.860.520	-0.0555
H	-1.084.653	21.385.946	42.199.253	-0.0543
H	0.778939	22.585.720	41.141.689	-0.0563
H	1.777.647	5.348.522	43.575.764	-0.0581
H	-0.487512	4.485.887	43.954.967	-0.0555
H	1.062.142	4.753.206	45.844.650	-0.0569
H	5.425.041	17.435.310	36.494.504	-0.0542
H	4.305.663	15.274.536	36.135.806	-0.0509
H	3.721.741	17.208.648	34.742.126	-0.0585
H	1.679.791	8.177.413	34.701.535	-0.0594
H	1.372.732	6.494.443	36.459.579	-0.0554
H	-0.305130	8.264.343	36.142.773	-0.0519
H	-7.478.142	8.593.047	39.355.700	-0.0571
H	-6.860.152	8.537.032	41.727.614	-0.0599

H	-8.565.821	10.116.700	40.948.525	-0.0573
H	8.059.436	17.587.844	43.710.921	-0.0576
H	6.925.835	18.954.415	45.410.566	-0.0575
H	6.190.409	19.039.929	43.074.588	-0.0593
H	-3.929.501	16.054.285	35.408.410	-0.0569
H	-6.241.623	16.181.508	36.224.207	-0.0563
H	-5.573.184	14.379.265	34.690.879	-0.0552
H	-7.848.319	15.832.105	43.388.905	-0.0574
H	-8.168.353	16.599.391	45.695.821	-0.0577
H	-7.621.929	18.214.596	43.924.897	-0.0568

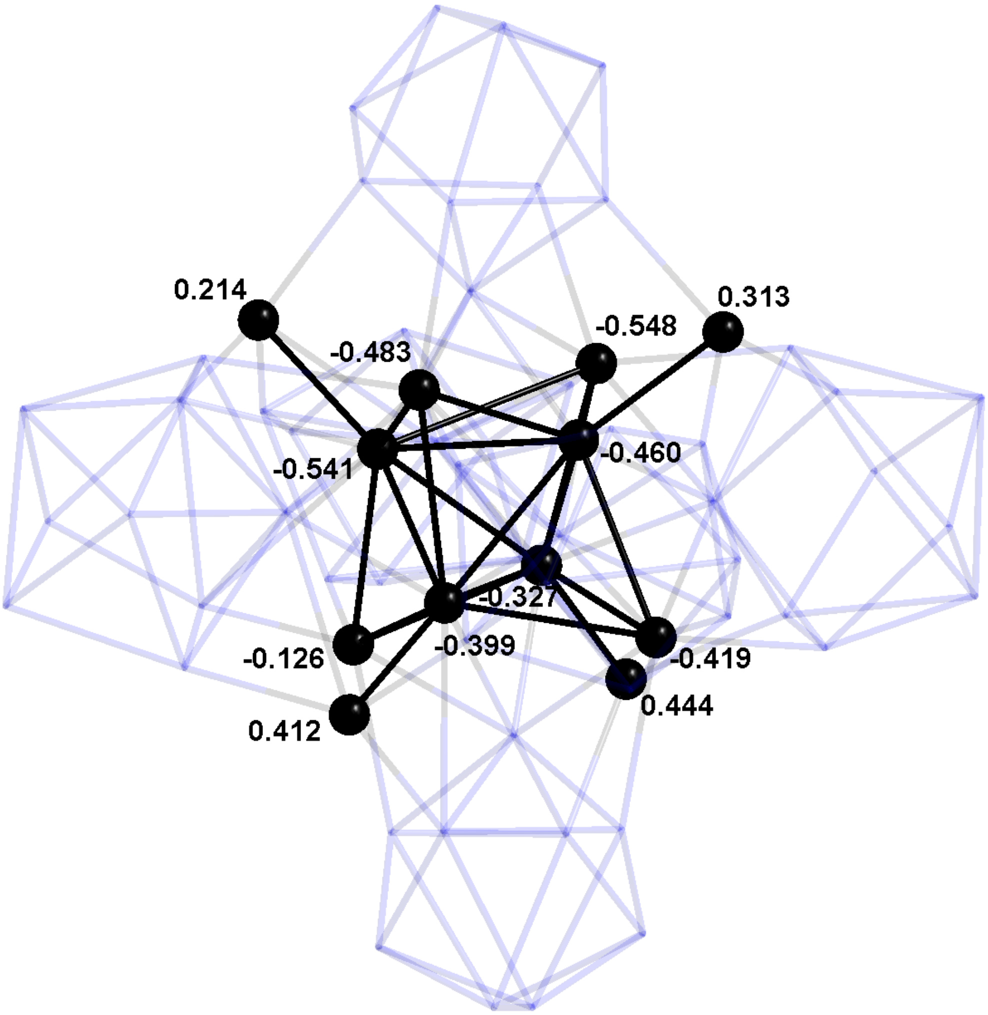

Figure S8: calculated partial charges for all Ag atoms.

8. References

[43] G. M. Sheldrick, *Acta Crystallogr.* **2008**, *A64*, 112−122.

[44] G. M. Sheldrick, *Acta Crystallogr., Sect. C: Struct. Chem.* **2015**, *C71*, 3−8.

[45] O. V. Dolomanov, L. J. Bourhis, R. J. Gildea, J. A. K. Howard, H. Puschmann, *J. Appl. Crystallogr.* **2009**, *42*, 339−341.

[46] K. Eichkorn, O. Treutler, H. Öhm, M. Häser, R. Ahlrichs, *Chem. Phys. Lett.* **1995**, *240*, 283 – 290.

[47] O. Treutler, R. Ahlrichs, *J. Chem. Phys.* **1995**, *102*, 346 – 354.

[48] J. P. Perdew, *Phys. Rev. B* **1986**, *33*, 8822 – 8824; A. D. Becke, *Phys. Rev. A* **1988**, *38*, 3098 – 3100.

[49] A. Schäfer, H. Horn, R. Ahlrichs, *J. Chem. Phys.* **1992**, *97*, 2571 – 2577.

[50] C. Steffen, K. Thomas, U. Huniar, A. Hellweg, O.Rubner, A. Schroer, *J. Comput. Chem.* **2010**, *31*, 2967 – 2970.

[51] E. R. Davidson, *J. Chem. Phys.* **1967**, *46*, 3320; K. R. Roby, *Mol. Phys.* **1974**, *27*, 81.

[52] R. Heinzmann, R. Ahlrichs, *Theor. Chim. Acta* **1976**, *42*, 33.

[53] C. Ehrhardt, R. Ahlrichs, *Theor. Chim. Acta* **1985**, *68*, 231.
